# New *eNeuro* Series: Improving Your Neuroscience

**DOI:** 10.1523/ENEURO.0048-24.2024

**Published:** 2024-03-12

**Authors:** Robert J. Calin-Jageman

Since Newton, the scientific literature has expanded exponentially, with an estimated growth rate of ∼5% per year since 1950 (Bornmann et al., 2021). This pace of growth is daunting. It means that half of *all* scientific papers were published in the last 15 years and that any scientist older than 48 has been alive for the publication of over 90% of the entire scientific literature. Of perhaps some comfort, the consistency of exponential growth in science means that *every* generation of scientist has looked back in awe (and despair?) at a burgeoning literature:

Science has always been modern; it has always been exploding into the population, always on the brink of expansive revolution. Scientists have always felt themselves to be awash in a sea of scientific literature …. (De Solla Price, 1963, p. 15)

If there is a corollary to the (so far) steady growth of science, it is steady improvement. Since Bacon’s original call to weed out human bias from our understanding of the natural world (1620), there has been a continual (and often contentious) evolution in how we apply the scientific method. Some notable milestones include the introduction of positive and negative controls (Pasteur, 1862), the coordination of experiments across multiple sites and centers (Dowling, 1975), the development of clinical trials (Lilienfeld, 1982), the integration of formal methods for statistical inference (Stigler, 1990), adoption of placebo and sham-treatment controls (Shorter, 2011), and the advent of clinical trial registries (Dickersin and Rennie, 2012). Scientists engage in a forever-war against false understanding, and this has perhaps been the key to the unparalleled success of the enterprise.

*eNeuro* is excited to announce a new article series to help readers carry on the never-ending battle to do the best science possible. This series, “Improving Your Neuroscience” will provide accessible, practical, authoritative tutorials on recent methodological innovations from which neuroscientists may benefit but which (so far) have uneven adoption. While an important focus of this series will be on statistical analyses, the goal is to consider, quite broadly, methodological practices that can help neuroscientists more regularly be “less wrong” (Rohrer and Murayama, 2023).

We believe the need for this series is quite strong. For example, for more than half a century, statisticians have been warning that scientists should not sequentially add samples to obtain statistical significance, as this seriously inflates the risk of spurious findings (Anscombe, 1954). In the United States, the National Institute of Health highlighted this issue almost a decade ago, including principled sample-size determination on its checklist of essential practices for conducting rigorous preclinical research (Moher et al., 2015). Still, change has been slow, and it is reasonable to infer that many (most?) neuroscientists are not completely sure how to best proceed (Carter et al., 2017). Similarly, *eNeuro* introduced the possibility of registered report in 2018 as a way to combat publication bias and to provide neuroscientists with an opportunity to propose and publish rigorous tests of important hypotheses. To date, however, only a handful of registered reports have been proposed, and there remains considerable trepidation about how to plan and propose a registered report despite funder encouragement (Crawford et al., 2023). A recent *eNeuro* paper has provided a comprehensive overview of this new approach and may help spur interest and adoption (Ellis, 2022). Other topics in clear need of attention include methods for ruling out an effect, Bayesian inference, comparison of effects (interactions), analysis of nested data, best practices for data and protocol sharing, achieving computational reproducibility, and techniques for error-proofing lab protocols (Strand, 2023).

While we are in the process of commissioning an initial batch of tutorials, we hope readers will submit suggestions for topics they would like to learn more about. Additionally, anyone who would be willing to write such a tutorial and share their knowledge is encouraged to email the eNeuro Editor in Chief, Christophe Bernard (christophe.bernard@univ-amu.fr), with a short cover letter explaining their proposal. In shaping this series, our guiding principles will be: pluralism (there are often multiple paths to strong research), breadth (topics broadly applicable will be prioritized over subfield-specific issues), and accessibility (while some theoretical background is always essential, tutorials in this series should focus primarily on neuroscience-specific examples and should provide practical advice on best practices for adoption).

The protean nature of our scientific endeavors is daunting, but equally invigorating. We are excited about the possibilities for this series to provide some respite, as well as encouragement to continue growing, adapting, and learning toward the best neuroscience possible.
